# Knockdown of LINC00662 represses AK4 and attenuates radioresistance of oral squamous cell carcinoma

**DOI:** 10.1186/s12935-020-01286-9

**Published:** 2020-06-16

**Authors:** Yangzong Chen, Chunchun Bao, Xiuxing Zhang, Xinshi Lin, Yimou Fu

**Affiliations:** Department of Chemotherapy and Radiotherapy, The Second Affiliated Hospital and Yuying Children’s Hospital of Wenzhou Medical University, Wenzhou, 325027 Zhejiang China

**Keywords:** Oral squamous cell carcinoma (OSCC), Radioresistance, LINC00662, hnRNPC, AK4

## Abstract

**Background:**

LncRNAs play crucial roles in the development of carcinomas. However, the investigation of LINC00662 in Oral squamous cell carcinoma (OSCC) is still elusive.

**Methods:**

qRT-PCR assay tested the expression levels of LINC00662, hnRNPC and AK4. With exposure to irradiation, CCK-8, colony formation, flow cytometry and western blot experiments, respectively determined the function of LINC00662 in the radiosensitivity of OSCC cells. Then RIP and western blot assays affirmed the interaction between hnRNPC protein and LINC00662 or AK4. Finally, rescue assays validated the regulation mechanism of LINC00662 in the radioresistance of OSCC.

**Results:**

In the present report, LINC00662 was overexpressed in OSCC and its silencing could alleviate radioresistance of OSCC. Furthermore, the interaction between hnRNPC protein and LINC00662 or AK4 was uncovered. Besides, LINC00662 regulated AK4 mRNA stability through binding to hnRNPC protein. To sum up, LINC00662 modulated the radiosensitivity of OSCC cells via hnRNPC-modulated AK4.

**Conclusion:**

The molecular mechanism of the LINC00662/hnRNPC/AK4 axis was elucidated in OSCC, which exhibited a promising therapeutic direction for patients with OSCC.

## Background

Oral squamous cell carcinoma (OSCC) is one of the most aggressive head and neck cancers all over the world [1]. Radiotherapy is a curative therapeutic method for OSCC [2], whereas the effect is still unsatisfactory due to the antergic radioresistance of OSCC [3]. Hence, a better understanding of the molecular regulation mechanism in OSCC was needed.

Long non‐coding RNAs (lncRNAs), a sort of non‐coding RNAs (ncRNAs), have more than 200 nucleotides in length and play crucial roles in carcinogenesis. Increasing evidence has indicated that aberrantly-expressed lncRNAs participate in cell proliferation, migration, invasion and even the radioresistance of human cancers [4–6]. For example, lncRNA NEAT1 promotes the radio-resistance of cervical cancer by miR-193b-3p/CCND1 axis [7]; lncRNA HOXC13-AS promotes nasopharyngeal carcinoma cell proliferation and invasion via regulating miR-383-3p/HMGA2 axis [8]; lncRNA CASC9 positively affects LIN7A expression by miR-758-3p to facilitate ovarian cancer [9]; lncRNA CASC2 downregulation promotes the postoperative local recurrence in early OSCC [10]. Long intergenic non-protein coding RNA 662 (LINC00662), located in chromosome 19, has been reported as an oncogene in colon cancer and hepatocellular carcinoma, influencing the cell growth, cell cycle and cell invasion [11, 12]. Besides, LINC00662 boosts cell invasion and cancer stem cell-like phenotypes in lung cancer [13]. LINC00662 promotes OSCC cell proliferation and migration [14]. In this study, we aimed to investigate the role and regulation mechanism of LINC00662 in OSCC cell radioresistance.

RNA-binding proteins usually function as major post-transcriptional regulators by RNA-binding activities [15]. Heterogeneous nuclear ribonucleoprotein C (C1/C2), also known as hnRNPC, belongs to the subfamily of ubiquitously expressed heterogeneous nuclear ribonucleoproteins (hnRNPs). In past researches, hnRNPC has been illustrated as pivotal mediators in human diseases, including carcinomas [16, 17]. Besides, hnRNPC is identified as survival-related splicing factor in OSCC [18]. However, the specific functional role and underlying mechanism of hnRNPC in OSCC was uncertain.

Adenylate kinase is a key enzyme in the high-energy phosphoryl transfer reaction in living cells. As an isoform of this enzyme, adenylate kinase 4 (AK4) has been demonstrated as a carcinogen, and plays crucial role in cancer cell resistance to radiation or drugs [19, 20]. For example, AK4 is a prognostic marker that facilitates metastasis in lung cancer via silencing ATF3, a transcription factor [21]. MiR-199a-3p targets AK4 to influence the multi-chemoresistance of osteosarcoma [19]. MiR-199a-3p targets AK4 to modulate the radioresistance of esophageal cancer cells [20]. The functional role of AK4 has never been investigated in OSCC.

The current study revealed that LINC00662 was high-expressed in OSCC. Mechanism experiments demonstrated that the silencing of LINC00662 improved the radiosensitivity of OSCC cells. Furthermore, hnRNPC protein was uncovered to interact with LINC00662 or AK4. Then, LINC00662 was confirmed to regulate AK4 mRNA stability through binding hnRNPC protein. Totally, upregulated LINC00662 reduced the radiosensitivity of OSCC cells via hnRNPC-modulated AK4 mRNA stability.

## Materials and methods

### Cell culture

Oral squamous cell carcinoma (CAL27, SCC-4, SCC-1, TU183 and SCC-9) and normal human oral keratinocyte (NHOK) cells were obtained from the cell bank of the Chinese Academy of Sciences (Shanghai, China). Cells were routinely incubated in Dulbecco’s modified Eagle medium (DMEM-Hyclone) mixed with 10% fetal bovine serum (FBS), 100 μg/ml streptomycin and 100U/ml penicillin under a 5% CO_2_ moist atmosphere at 37 °C.

### Cell transfection

The full-length sequence of hnRNPC or AK4 was subcloned into the pcDNA3.1 plasmid (pcDNA3.1-hnRNPC or pcDNA3.1-AK4; Invitrogen,Carlsbad, CA, USA). Knockdown of LINC00662 (sh-LINC00662#1 or sh-LINC00662#2) or hnRNPC (sh-hnRNPC) was also achieved by Invitrogen. The transfection was conducted using Lipofectamine 2000 (Invitrogen, Carlsbad, CA, USA) following the instructions of the manufacturer. The sequences of relevant shRNAs are as follows: sh-NC, 5′-GAGCCTGGATGATACATGATGC-3′; sh-LINC00662#1, 5′-CTCTTCTATCGAACCGGCCCGC-3′; sh-LINC00662#2, 5′-AGTACTGAACACGGGTTTCAAA-3′; sh-NC, 5′-AGCGAAGGGTCAGGAGAAAGAT-3′; sh-hnRNPC#1, 5′-CGCTCTCCCCCACACCCTCTCT-3′; sh-NC, 5′-AAGTGTAAGAGATGAAAGTAGA-3′; sh-AK4#1, 5′-GCTCTGCGTCTGGTGTGCAACG-3′.

### Quantitative real time PCR (qRT-PCR)

TRIzol (Life Technologies, Carlsbad, CA, USA) was adopted to extract total RNA from OSCC cells and RNA purity was assessed using spectrophotometer (Bio-Rad, Hercules, CA, USA). The Prime-Script miRNA cDNA Synthesis Kit (TaKaRa, Tokyo, Japan) to synthesize cDNA using 1 μg of total RNA according to manufacturer’s protocol. The SYBR^®^ Premix Ex TaqTM reagent (TaKaRa, Dalian, China) was employed for qPCR analysis on an ABI PRISM 7500 real-time PCR system (Applied Biosystems, Foster City, CA, USA). GADPH was the internal control. The 2^−ΔΔCT^ method was adopted to quantify the results. The primer sequences were as follows: LINC00662 forward: 5′-CACGCTTCTGAAACTGGTGT-3′, and reverse: 5′-TGTACAGCCTGGTGACAGAG-3′; AK4 forward: 5′-TGGATTCACCCTCCTAGCGGAA-3′, and reverse: 3′-CTGTCTTAGCCTGGCAGCAACT-5′; hnRNPC forward: 5′-TGGGCTGCTCTGTTCATAAGGG-3′, and reverse: 5′-CTCGGTTCACTTTTGGCTCTGC-3′; GAPDH forward: 5′-GCACCGTCAAGGCTGAGAAC-3′, and reverse: 5′-TGGTGAAGACGCCAGTGGA-3′.

### Cell counting kit-8 (CCK-8) assay

CCK-8 assay measured the proliferation capacity of cells. Transfected cells were seeded into 96-well culture dishes with a density of 3 × 10^3^ cells/well for 12-h culture with or without 4Gy radiation treatment at the dose rate of 200 cGy/min using ^137^Cs γ-ray source (Atomic Energy of Canada Ltd, Mississauga, Ontario, Canada). Then, cell counting kit-8 (10 μl/well, Dojindo, Kumamoto, Japan) was added according to manufacturer’s protocol, followed by incubation at 37 °C for another 3 h. Absorbance at 450 nm was detected by the MRX II microplate reader (Dynex, Chantilly, VA, USA).

### Colony formation assay

Transfected OSCC cells were inoculated into a 6-well culture dish (500 cells/well) and were exposed to several radiation doses (0, 2, 4 and 8Gy). All cells were cultured for 2 weeks, then washed by PBS, fixed by 10% formaldehyde, and stained by 0.1% Crystal Violet (Sigma, U.S.A.). The number of colonies more than 50 cells was counted, based on which cell survival fraction (plating efficiency (PE); SF = each dose of PE/non-irradiated PE × 100%) was calculated.

### Flow cytometry of cell apoptosis and cell cycle

For cell apoptosis analysis, cells were inoculated into six-well plates and transfected with sh-NC or sh-LINC00662#2 for 48 h, and then treated with a 4Gy irradiation for 24 h. OSCC cells were washed twice by PBS, centrifuged to isolate the debris and then re-suspended in binding buffer at a concentration of 1 × 10^6^ cells per ml. Cells (1 × 10^6^). Afterwards, cells were dyed with Annexin V/propidium iodide (PI) reagents (KeyGen Biotech, Nanjing, China) according to the instructions of the manufacturers. After the addition of binding buffer (0.4 ml), cells were examined by a BD FACSCanto II flow cytometer (BD Biosciences, San Jose, CA, USA). As for cell cycle analysis, cells were co-cultured in 6-well plates (3 × 10^5^ cells/well) with propidium iodide (PI) staining buffer (Dojindo Molecular Technologies, Inc) and subjected to flow cytometer (BD Biosciences). The proportion of cells in G0/G1, S, or G2/M phases was analyzed with the Cell Quest Pro acquisition software (BD Biosciences).

### Flow cytometry of transfection efficiency

The indicated cells (5 × 10^5^) were seeded into each well of 24-well plates and cultured with complete DMEM for 24 h prior to transfection. The cells treated alone with pSEB pooled plasmids containing transfection plasmids were named as group I; with pSEB plasmids with ultrasound were named as group II; with the lipid microbubble loaded with transfection plasmids were named as group III; with ultrasound and the lipid microbubble loaded with transfection plasmids were named as group IV; with non-plasmid control were named as group V. The Lipofection group (Lipo) was used for comparing transfection efficiency. Cells of group II and IV were ultra-sonicated with 1 MHz of radiation frequency, pulse wave and 0.5 W/cm^2^ of sound intensity for 30 s. After that, the transfected cells were harvested after 48 h for flow cytometry to detect transfection efficiency.

### Transwell assays

Cell migratory ability was evaluated with the use of the Transwell polycarbonate membrane inserts (Millipore, Billerica, MA, USA). The transfected CAL27 and SCC-4 cells (1 × 10^5^) were planted into the upper chamber of the insert. 10% FBS was used to supplement cell-free medium in the low chamber at 37 °C for 1 day. Cells migrating through the membranes were cultured in 4% paraformaldehyde and stained with 0.1% crystal violet. For cell invasion assay, 1 × 10^5^ cells in sterile medium were seeded into the upper chamber with Matrigel (Sigma-Aldrich, St. Louis, MO, USA). Both migrated and invaded cells were observed and counted under an inverted light microscope (Leica Microsystems, Wetzlar, Germany) at a magnification of ×200.

### Knockout of LINC00662 or hnRNPC by CRISPR/Cas9

The CRISPR/Cas9 genome editing system was applied for generating the LINC00662 or hnRNPC knockout cells. The two sgRNAs targeting LINC00662 or hnRNPC were individually transfected into cells and Cas9-EGFP vector. The pCRISPR-LvSG03 for sgRNAs and CP-LvC9NU-02 for construction of Cas9-EGFP were all procured from Genecopoeia (Rockville, MD, USA). Cas9-expressing cells were established before co-infection of two sgRNAs. The genomic deletions of isolated single colonies were detected using T7 endonuclease I cleavage and PCR, and determined with sequencing after cloning the relative PCR products to T vector (Takara).

### Bioinformatics prediction

starBase v2.0 (http://starbase.sysu.edu.cn/starbase2/browseClipSeq.php) was employed to predict the RNA-binding proteins of LINC00662 or AK4. In the Protein-RNA column, we chose protein-lncRNA interactions and protein-mRNA interactions parts, respectively. Among the putative LINC00662-binding proteins and AK4-binding proteins, we selected hnRNPC, one of the top ten RNA-binding protein candidates according to starBase v3.0 (http://starbase.sysu.edu.cn/), for further studies.

### RNA immunoprecipitation (RIP) assay

For RNA immunoprecipitation (RIP) assay, a Magna RIP Kit (Millipore, Billerica, MA, USA) was adopted according to the manufacturer’s protocols. The whole cell lysate was incubated in RIP buffer where magnetic beads were absorbed by anti-hnPNRC (ab133607; 1:100; Abcam, Cambridge, MA, USA) or anti-IgG (ab218427; 1:100; CST, Boston, MA, USA). IgG was a normalization control. After the digestion of proteinase K, co-precipitated RNAs including LINC00662 and AK4 were harvested, purified and detected by qRT-PCR.

### RNA pull-down assay

LINC00662 or AK4 sense was named as LINC00662-WT or AK4-WT, compared with LINC00662 or AK4 antisense (LINC00662-Mut or AK4-Mut). LINC00662-WT, LINC00662-Mut, AK4-WT and AK4-Mut were in vitro transcribed by TranscriptAid T7 High Yield Transcription Kit (Thermo Fisher Scientific, Inc., Waltham, MA, USA) and subsequently biotin‐labelled through the Biotin RNA Labelling Mix (Roche) and T7 RNA polymerase (Roche) as instructed. After the incubation with DNase I (Takara), the RNeasy Mini Kit (Qiagen, Valencia, CA, USA) was utilized to purify the transcribed RNAs. Then, 3 μg of biotinylated RNAs were grown for 1 h at 25 °C in mixture with 1 mg whole cell lysates. Then, the complexes were extracted via streptavidin-conjugated Dynabeads (Invitrogen). Eventually, the pull-down materials were confirmed by western blot.

### Western blot

CAL27 and SCC-4 cells were cultured in RIPA buffer with protease inhibitor cocktails (AMRESCO), lysed for 30 min with brief vortexing every 10 min. Then centrifuge the lysates for 15 min at 4 °C and detect protein concentrations using the BCA Protein Assay Reagent (Pierce, Appleton, Wisconsin, USA). Post separation of 40 μg protein by sodium dodecyl sulfate polyacrylamide gel electrophoresis (SDS-PAGE), the lysate was transferred to nitrocellulose membranes. The membranes were incubated with primary antibodies against cleaved PARP (ab32064; Abcam), PARP (ab74290; Abcam), cleaved caspase-3 (ab2302; Abcam), caspase-3 (ab13847; Abcam), hnRNPC (ab97541; Abcam), AK4 (ab232888; Abcam) or GAPDH (sc-32233; Santa Cruz Biotechnology, Santa Cruz, CA, USA) at 4 °C overnight. After washing, the membrane was cultured with horseradish peroxidase-conjugated (HRP) secondary antibody (Santa Cruz Biotechnology, Inc., Dallas, TX, USA) at room temperature for 2 h. Finally, the signals were estimated by enhanced fluorescence as suggested.

### Statistical analysis

All statistical analyses were conducted via SPSS 18.0 (SPSS, Inc., Chicago, IL, USA) and GraphPad Prism software (GraphPad, Inc., La Jolla, CA, USA). The data were indicated as the mean ± standard deviation. Statistical significance analysis of two or multiple groups was performed using Student’s t-test or ANOVA. P value less than 0.05 was accepted as statistically significant. Each assay was independently carried out in triplicate (Additional file 1).

## Results

### LINC00662 silencing attenuated radioresistance of OSCC cells

**Fig. 1:**
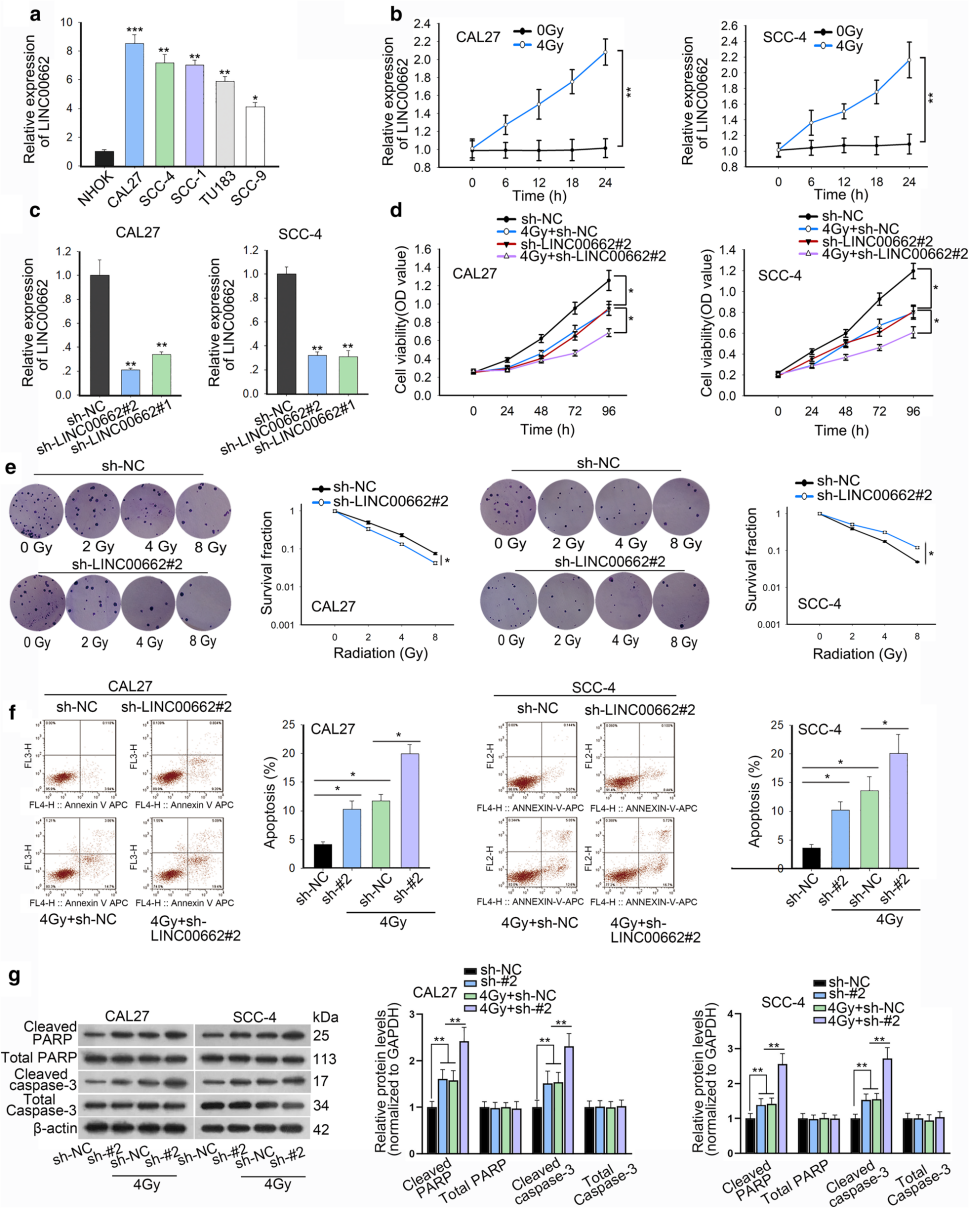
LINC00662 attenuated radioresistance of OSCC cells. **a** qRT-PCR analysis of LINC00662 expression in OSCC (CAL27, SCC-4, SCC-1, TU183 and SCC-9) and normal human oral keratinocytes (NHOK) cell lines. **b** LINC00662 expression in CAL27 and SCC-4 cells under 4Gy irradiation was tested by qRT-PCR. **c** The expression of LINC00662 by transfection of sh-LINC00662#1 or sh-LINC00662#2 in CAL27 and SCC-4 cells was also measured by qRT-PCR. **d** CCK-8 assay was performed to examine cell viability of sh-LINC00662#2 transfected CAL27 and SCC-4 cells under 0 or 4Gy radiation, compared with relative control groups. **e** Survival fractions of sh-LINC00662#2 treated CAL27 and SCC-4 cells at the indicated doses of 0, 2, 4 and 8Gy radiation were respectively determined by colony formation assay. **f** Flow cytometry analysis of cell apoptosis in CAL27 and SCC-4 cells with LINC00662 down-regulation after 0 or 4Gy irradiation treatment. **g** Under 0 or 4Gy irradiation, cleaved PARP, cleaved caspase-3, total PARP and caspase-3 levels in CAL27 and SCC-4 cells with LINC00662 silencing were detected through western blot. *P < 0.05, **P < 0.01, and ***P < 0.001

To explore the expression profiles of LINC00662 in OSCC, we firstly tested its expression in OSCC cell lines (CAL27, SCC-4, SCC-1, TU183 and SCC-9) and the primary normal human oral keratinocytes (NHOK) cell lines. qRT-PCR revealed that the expression of LINC00662 was relatively up-regulated in OSCC cells (Fig. 1a). The sensitivity of SCC-4 and CAL27 cells to radiation was significantly lower than that of normal NHOK cell (Additional file 2: Figure S1A). Among the incubated OSCC cell lines, CAL27 and SCC-4 cells with the highest level of LINC00662 were chosen for subsequent experiments. LINC00662 expression was further enhanced in CAL27 and SCC-4 cells under 4Gy irradiation at a time dependent manner, compared to no irradiation exposure (Fig. 1b). In order to investigate the effect of LINC00662 on the radioresistance of OSCC, sh-LINC00662#1 or sh-LINC00662#2 was transfected into CAL27 and SCC-4 cells. LINC00662 expression was significantly reduced by transfection of sh-LINC00662#1/2, as examined by qRT-PCR (Fig. 1c and Additional file 1A). Here, since sh-LINC00662#2 exhibited higher transfection efficiency than sh-LINC00662#1, the knockdown effect of sh-LINC00662#2 was better than that of sh-LINC00662#1, and we selected sh-LINC00662#2 for later assays. CCK-8 assay illustrated that 4Gy irradiation or LINC00662 repression inhibited cell proliferation of CAL27 and SCC-4 cells, and this effect was strengthened under the co-treatment of 4Gy irradiation and LINC00662 knockdown (Fig. 1d). Colony formation assay indicated that there was a declining trend of cell survival fraction with the increased irradiation doses. LINC00662 down-regulation could also restrain survival fraction in both CAL27 and SCC-4 cells. Importantly, combination of irradiation and LINC00662 silencing markedly reinforced the declining trend of cell survival fraction (Fig. 1e). Flow cytometry cell cycle analysis showed that radiation or LINC00662 silence arrested cell cycle at G0/G1 phrase. Silenced LINC00662 further increased proportion of cells at G0/G1 phrase caused by 4Gy radiation (Additional file 2: Figure S1B). In flow cytometry assay, apoptosis was obviously enhanced by radiation exposure or by transfection of sh-LINC00662#2. Combination of radiation exposure and the knockdown of LINC00662 further enhanced cell apoptosis rates of CAL27 and SCC-4 cells (Fig. 1f). Poly ADP-ribose polymerase (PARP) is a kind of DNA repairing enzyme and also an apoptosis-related protein [22]. Western blotting demonstrated that the expression level of cleaved-PARP and cleaved caspase-3 decreased after 4Gy irradiation or silence of LINC00662. Such effect was further significantly boosted through the coexistence of radiation and LINC00662 inhibition. In the meanwhile, expression of total PARP or caspase-3 showed no changes to 4Gy irradiation or silence of LINC00662 (Fig. 1g). Besides, cell motility was also assessed via transwell assays. The results exhibited that LINC00662 silencing further aggravated the suppressive effects of 4Gy irradiation on cell migration and invasion (Additional file 2: Figure S1C and D).

Moreover, we constructed the LINC00662-KO plasmid to knock out LINC00662 expression. LINC00662 expression was significantly knocked out by transfection of LINC00662-KO (Additional file 3: Figure S2A). All the functional assays demonstrated that LINC00662-KO significantly enhanced OSCC cell sensitivity to radiation exposure (Additional file 3: Figure S2B–H).

In conclusion, all these data revealed that LINC00662 served as an oncogene in OSCC cells, and LINC00662 knockdown or knockout improved radiosensitivity of OSCC cells.

### LINC00662 bound with hnRNPC protein and had no regulatory influence on hnRNPC

**Fig. 2:**
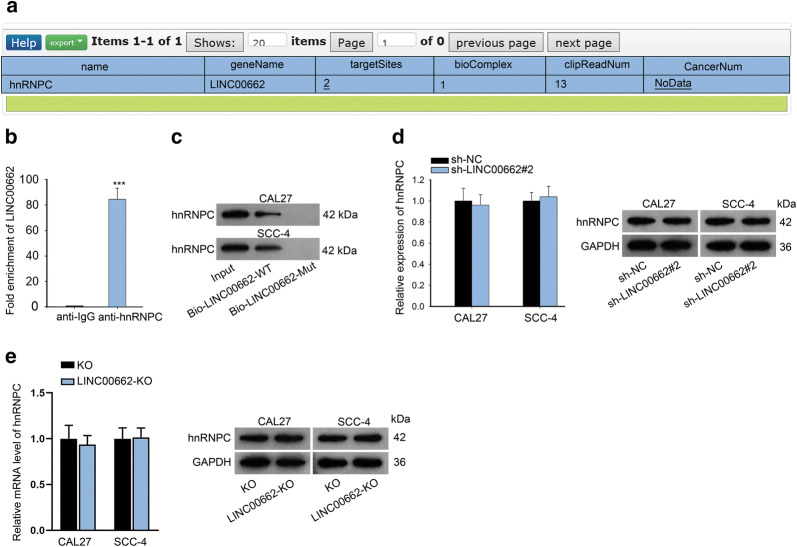
LINC00662 bound with hnRNPC protein and had no regulatory influence on hnRNPC. **a** The predicted binding between LINC00662 and hnRNPC was obtained from starBase. **b**–**c** RIP and western blot assays validated the interaction of LINC00662 and hnRNPC protein. **d** hnRNPC expression had no alteration when LINC00662 was knocked down, as estimated by qRT-PCR and western blot. **e** qRT-PCR and western blot revealed influence of LINC00662 knockout on hnRNPC expression. ***P < 0.001

Past researches have unveiled that RNA-binding proteins (RBPs) can interact with lncRNAs in tumors [23]. From starBase, we discovered that LINC00662 potentially bound with hnRNPC protein (Fig. 2a). RIP and western blot assays were performed to confirm the combination of LINC00662 with hnRNPC protein. In RIP experiment, the mixtures immunoprecipitated by anti-hnRNPC exhibited an obvious enrichment of LINC00662 (Fig. 2b). The western blot assay demonstrated that LINC00662 interacted with hnRNPC protein (Fig. 2c). Moreover, the mRNA and protein level of hnRNPC exhibited no change in response to sh-LINC00662#2 group, compared with control group (Fig. 2d). Besides, knockout of LINC00662 had no influences on the mRNA and protein level of hnRNPC (Fig. 2e). These results proved that LINC00662 bound to hnRNPC protein and had no regulatory influence on hnRNPC.

### hnRNPC bound with AK4 mRNA to stabilize AK4 mRNA

**Fig. 3:**
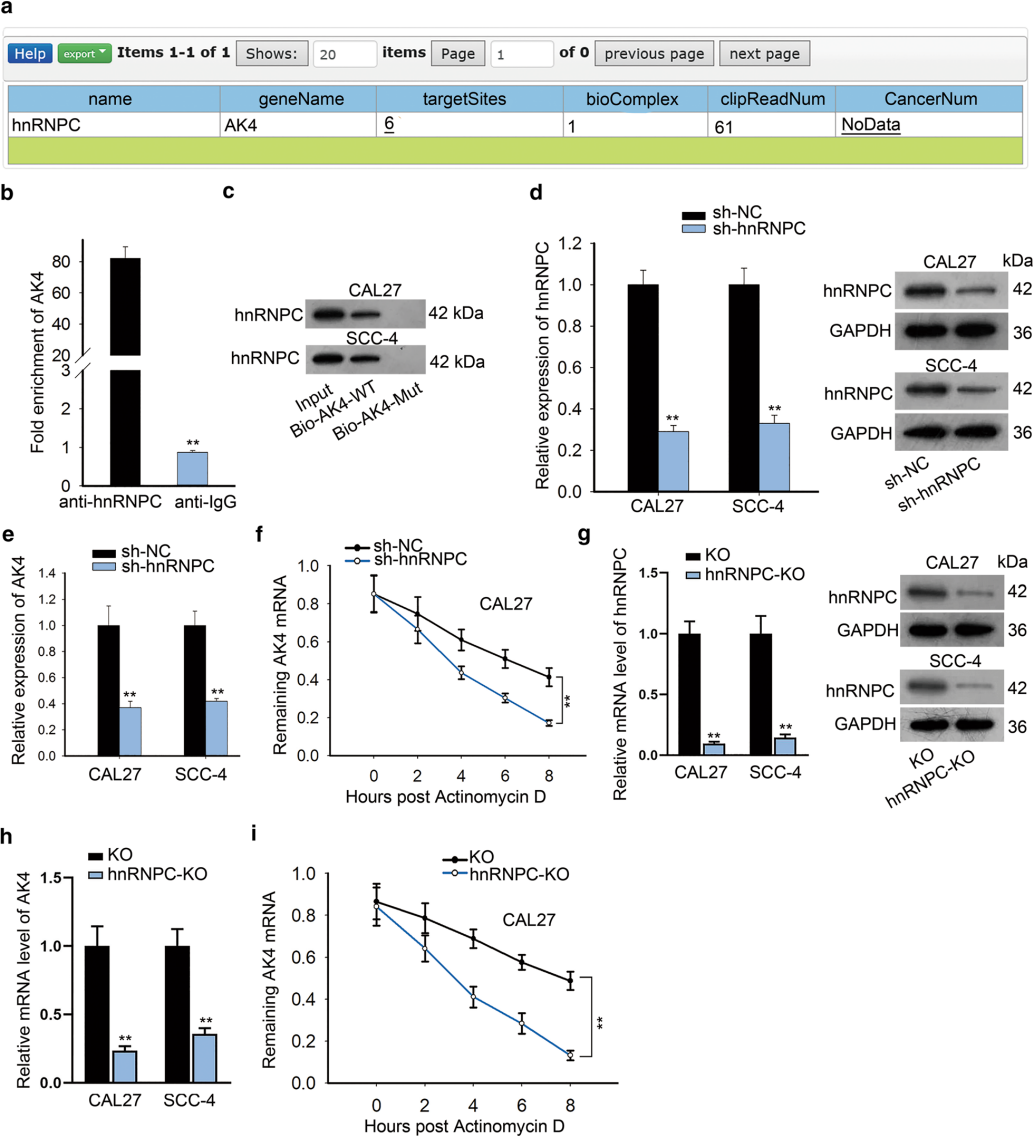
hnRNPC bound with AK4 mRNA to stabilize AK4 mRNA. **a** The putative binding capacity between hnRNPC and AK4 was predicted from starBase. **b**, **c** The combination of hnRNPC with AK4 was confirmed by RIP and western blot assays. **d** qRT-PCR and western blot evaluated hnRNPC expression in sh-hnRNPC transfected CAL27 and SCC-4 cells. **e** qRT-PCR detection of AK4 level in CAL27 and SCC-4 cells with the transfection of sh-hnRNPC. **f** Under actinomycin D treatment, the mRNA stability of AK4 was assessed through qRT-PCR by knockdown of hnRNPC. **g** qRT-PCR and western blot evaluated hnRNPC expression in hnRNPC-KO transfected CAL27 and SCC-4 cells. **h** qRT-PCR revealed AK4 expression by knockout of hnRNPC. **i** Under actinomycin D treatment, the mRNA stability of AK4 was assessed through qRT-PCR by knockout of hnRNPC. **P < 0.01

RNA-binding proteins are also reported to interact with mRNAs and further stabilize their mRNA expression levels [24]. From starBase, we found the potential combination of AK4 with hnRNPC (Fig. 3a). As AK4 was reported to modulate the radioresistance of cancer cells [20], we guessed that AK4 might participate in LINC00662-mediated radioresistance in OSCC cells. RIP assay illustrated that the compounds precipitated by anti-hnRNPC exhibited abundant enrichment of AK4 (Fig. 3b). RNA pull-down and western blot assays further verified that hnRNPC protein bind with bio-AK4-WT (Fig. 3c). Furthermore, we probed the effects of hnRNPC on AK4. The expression level of hnRNPC was markedly knocked down at both mRNA and protein level after sh-hnRNPC was stably transfected into CAL27 and SCC-4 cells (Fig. 3d and Additional file 1B). As a result, AK4 expression was significantly reduced by knockdown of hnRNPC (Fig. 3e), as measured by qRT-PCR. Besides, qRT-PCR displayed that the remaining mRNA of AK4 was reduced when actinomycin D was added, and this phenomenon was aggravated by the knockdown of hnRNPC, showing the positive regulation of hnRNPC on the stability of AK4 mRNA (Fig. 3f). After that, we constructed hnRNPC-KO plasmids to evaluate the effects of hnRNPC knockout on AK4. hnRNPC expression was significantly knocked out at both mRNA and protein level after stable transfection of hnRNPC-KO (Fig. 3g). Knockout out of hnRNPC remarkably reduced AK4 expression (Fig. 3h). Also, knockout out of hnRNPC aggravated degradation of AK4 caused by actinomycin D (Fig. 3i). Collectively, hnRNPC protein interacted with AK4 mRNA and further stabilized AK4 mRNA.

### LINC00662 promoted AK4 mRNA stability through binding to hnRNPC protein

**Fig. 4:**
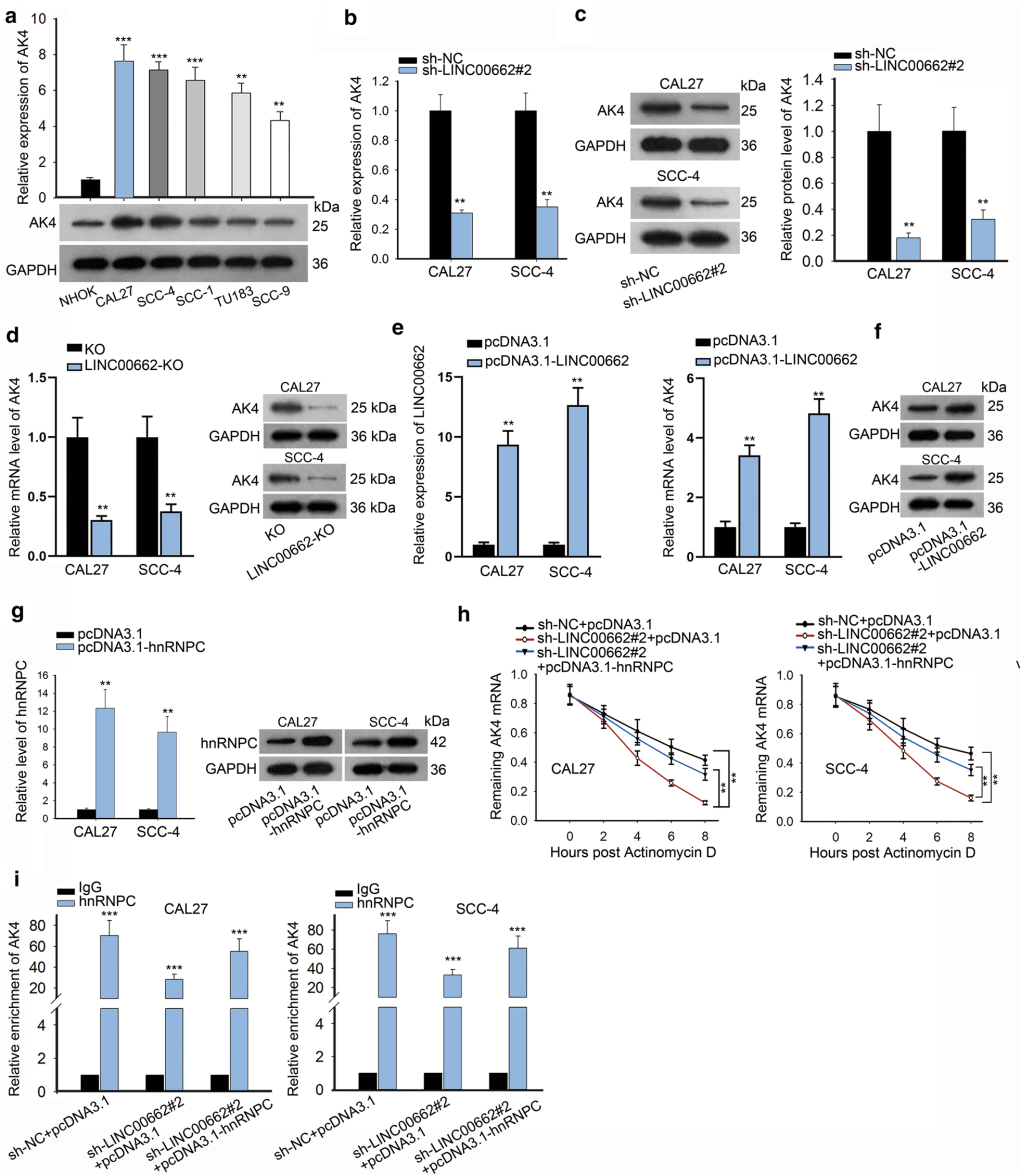
LINC00662 promoted AK4 mRNA stability through binding to hnRNPC protein. **a** AK4 expression levels in OSCC cell lines and NHOK were examined separately by qRT-PCR and western blot. **b**, **c** qRT-PCR and western blot results of the mRNA and protein levels of AK4 in sh-LINC00662#2 transfected CAL27 and SCC-4 cells. **d** qRT-PCR and western blot revealed the mRNA and protein levels of AK4 in LINC00662-KO transfected CAL27 and SCC-4 cells. **e** qRT-PCR revealed expression of LINC00662 and AK4 in pcDNA3.1-LINC00662 transfected CAL27 and SCC-4 cells. **f** Western blot assay revealed expression of AK4 by LINC00662 overexpression. **g** hnRNPC expression in CAL27 and SCC-4 cells transfected with pcDNA3.1-hnRNPC was measured through qRT-PCR and western blot assay. **h** With the addition of actinomycin D, qRT-PCR assessed the mRNA stability of AK4 in CAL27 and SCC-4 cells under the co-transfection of sh-LINC00662#2 and pcDNA3.1, or co-transfection of sh-LINC00662#2 and pcDNA3.1-hnRNPC. **i** RIP assays were carried out to test the impacts of LINC00662 and hnRNPC on the combination between hnRNPC protein and AK4. **P < 0.01 and ***P < 0.001

LncRNAs have been revealed to interact with RBPs and then regulate the stability of their target genes [25]. Hence, we supposed that LINC00662 modulated AK4 mRNA stability via binding to hnRNPC protein. qRT-PCR and western blot analysis revealed the up-regulated expression of AK4 in OSCC cell lines, in comparison with NHOK cell lines (Fig. 4a). By qRT-PCR and western blot analysis, AK4 expression was overtly downregulated at both mRNA and protein levels on account of LINC00662 inhibition (Fig. 4b, c). Also, knockout of LINC00662 dramatically reduced AK4 expression at both mRNA and protein level (Fig. 4d). Then, we detected the influence of LINC00662 overexpression on AK4 expression. Transfection of pcDNA3.1-LINC00662 significantly enhanced expression of AK4 at both mRNA and protein level (Fig. 4e–f and Additional file 1C) Thereafter, pcDNA3.1-hnRNPC was transfected into CAL27 and SCC-4 cells. Transfection of pcDNA3.1-hnRNPC resulted in the overexpression of hnRNPC, as determined by qRT-PCR and western blot (Fig. 4g and Additional file 1D). Finally, qRT-PCR demonstrated that in both CAL27 and SCC-4 cells, actinomycin D inhibited the RNA synthesis of AK4, which was exacerbated after LIN00662 was downregulated but restored when hnRNPC was upregulated (Fig. 4h). Furthermore, RIP experiment was performed to explore whether LINC00662 served as a scaffold for the binding between hnRNPC and AK4. The results displayed that the binding of hnRNPC with AK4 was blocked by sh-LINC00662#2 but recovered by pcDNA3.1-hnRNPC (Fig. 4i). Taken together, we concluded that LINC00662 recruited hnRNPC protein to increase AK4 expression.

### LINC00662 modulated the radiosensitivity of OSCC cells via AK4

**Fig. 5:**
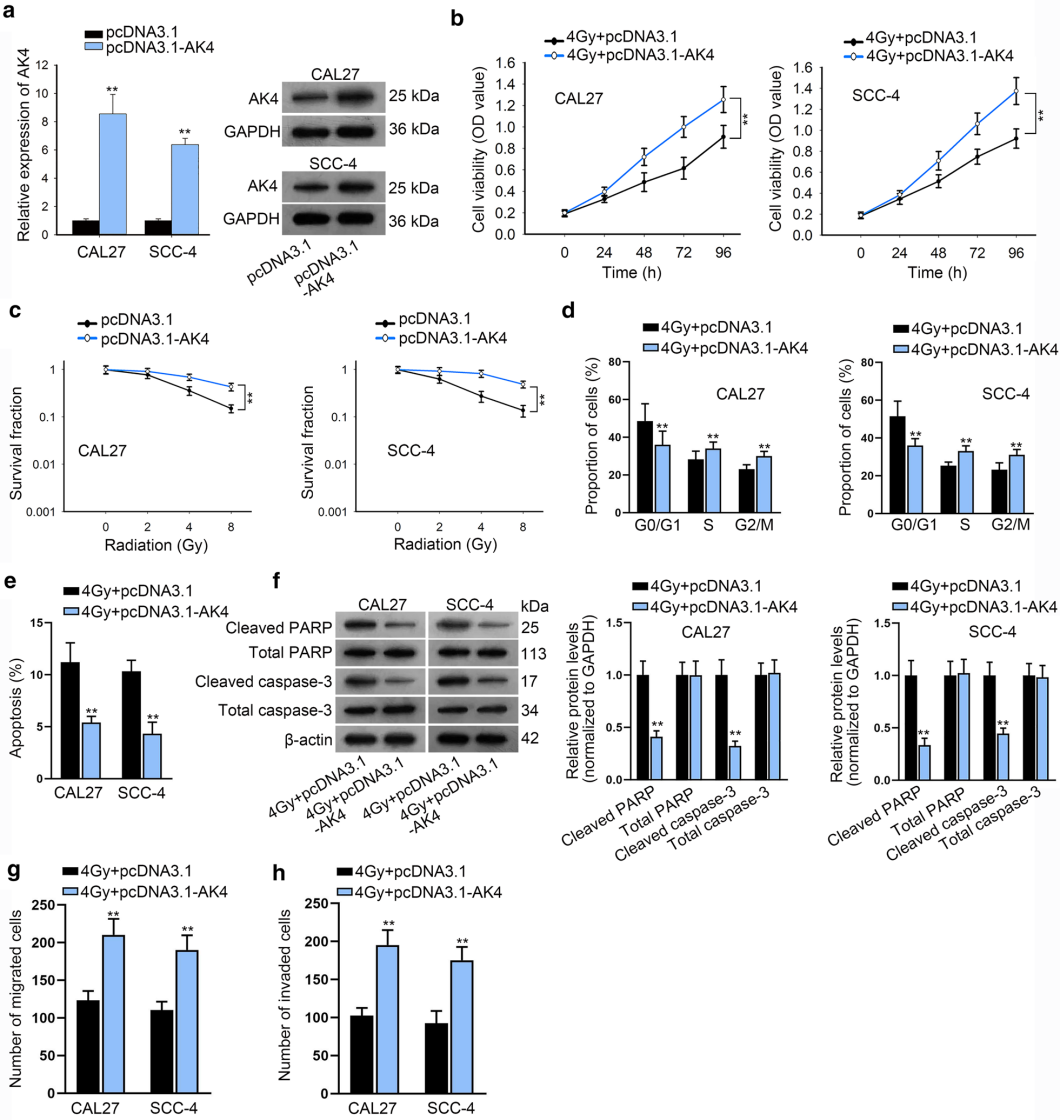
AK4 overexpression enhanced CAL27 and SCC-4 cell resistance to radiation. **a** The overexpression efficacy of AK4 in CAL27 and SCC-4 cells was detected by qRT-PCR and western blot assay. **b** CCK-8 assay was performed to examine cell viability of pcDNA3.1-AK4 transfected CAL27 and SCC-4 cells under 4Gy radiation. **c** Survival fractions of pcDNA3.1-AK4 treated CAL27 and SCC-4 cells at 4Gy radiation were determined by colony formation assay. **d**–**h** Cell cycle, apoptosis, migration and invasion abilities were tested through flow cytometry, western blot and transwell assays in CAL27 and SCC-4 cells transfected with pcDNA3.1-AK4. **P < 0.01, and ***P < 0.001

Rescue assays were performed to affirm the function of LINC00662/hnRNPC/AK4 axis in OSCC. We firstly evaluated function of AK4 overexpression in OSCC cell radioresistance. In qRT-PCR assay, AK4 expression was significantly up-regulated at approximately 8.5-fold increase in CAL27 cells and approximately 6.2-fold increase in SCC-4 cells by transfection of pcDNA3.1-AK4; the overexpression efficiency of AK4 was also verified in the following western blot assay (Fig. 5a and Additional file 1E). The gain of function assays depicted that AK4 overexpression enhanced cell radioresistance by promoting cell viability (Fig. 5b), proliferation (Fig. 5c), inhibiting cell cycle arrest (Fig. 5d) and apoptosis (Fig. 5f), facilitating migration (Fig. 5g) and invasion (Fig. 5h).

**Fig. 6:**
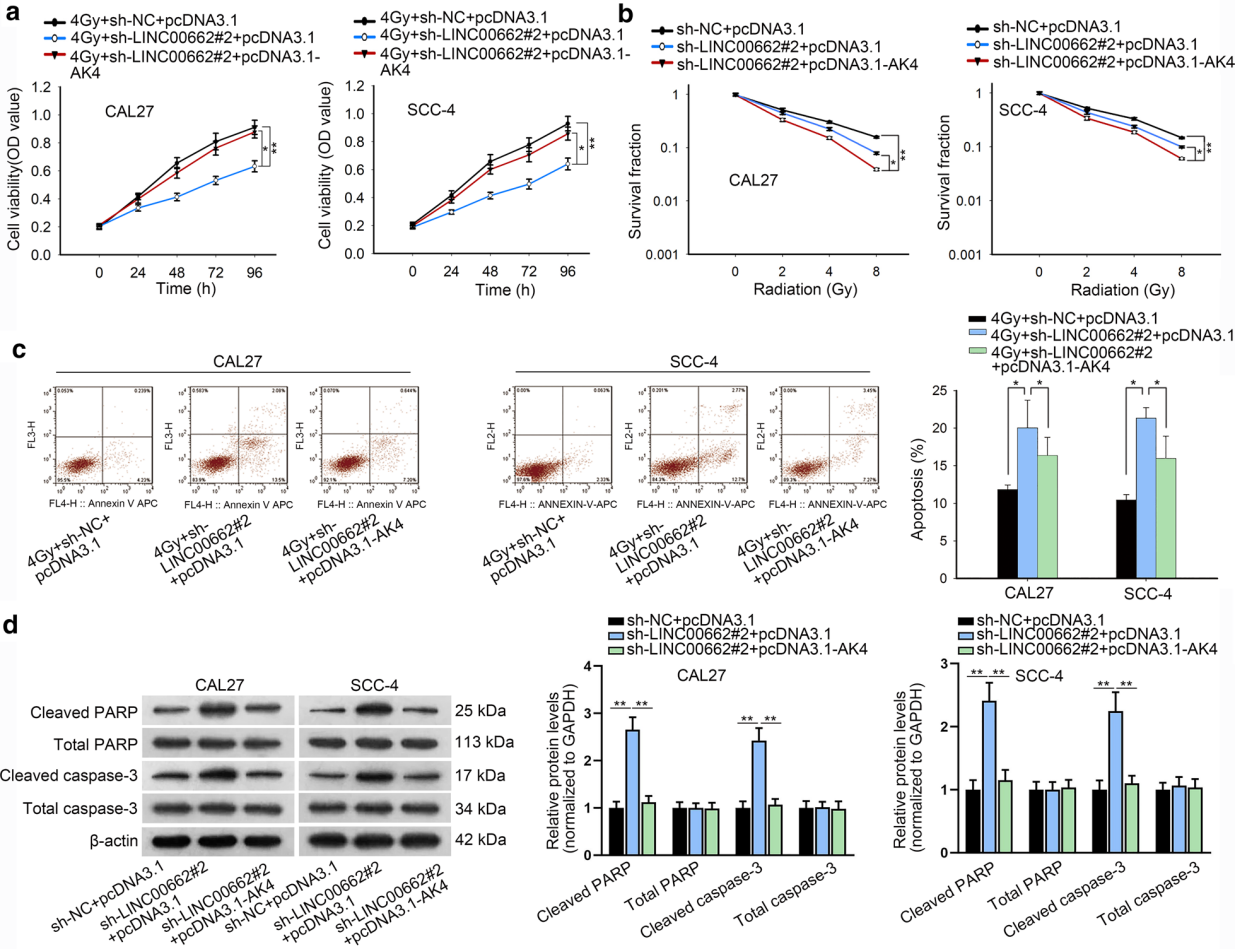
LINC00662 promoted the radiosensitivity of OSCC cells via AK4. **a** CCK-8 experiment evaluated cell proliferation of CAL27 and SCC-4 cells under 4Gy irradiation with AK4 overexpression to rescue silenced LINC00662. **b** In colony formation assay, survival fraction of CAL27 and SCC-4 cells was determined at the indicated doses of 0, 2, 4 and 8Gy irradiation with AK4 overexpression to rescue silenced LINC00662. **c** Flow cytometry result of cell apoptosis in CAL27 and SCC-4 cells with AK4 overexpression to rescue silenced LINC00662. **d** Protein levels of cleaved PARP, cleaved caspase-3, total PARP and caspase-3 in CAL27 and SCC-4 cells were examined through western blot with AK4 overexpression to rescue silenced LINC00662. *P < 0.05 and **P < 0.01

**Fig. 7:**
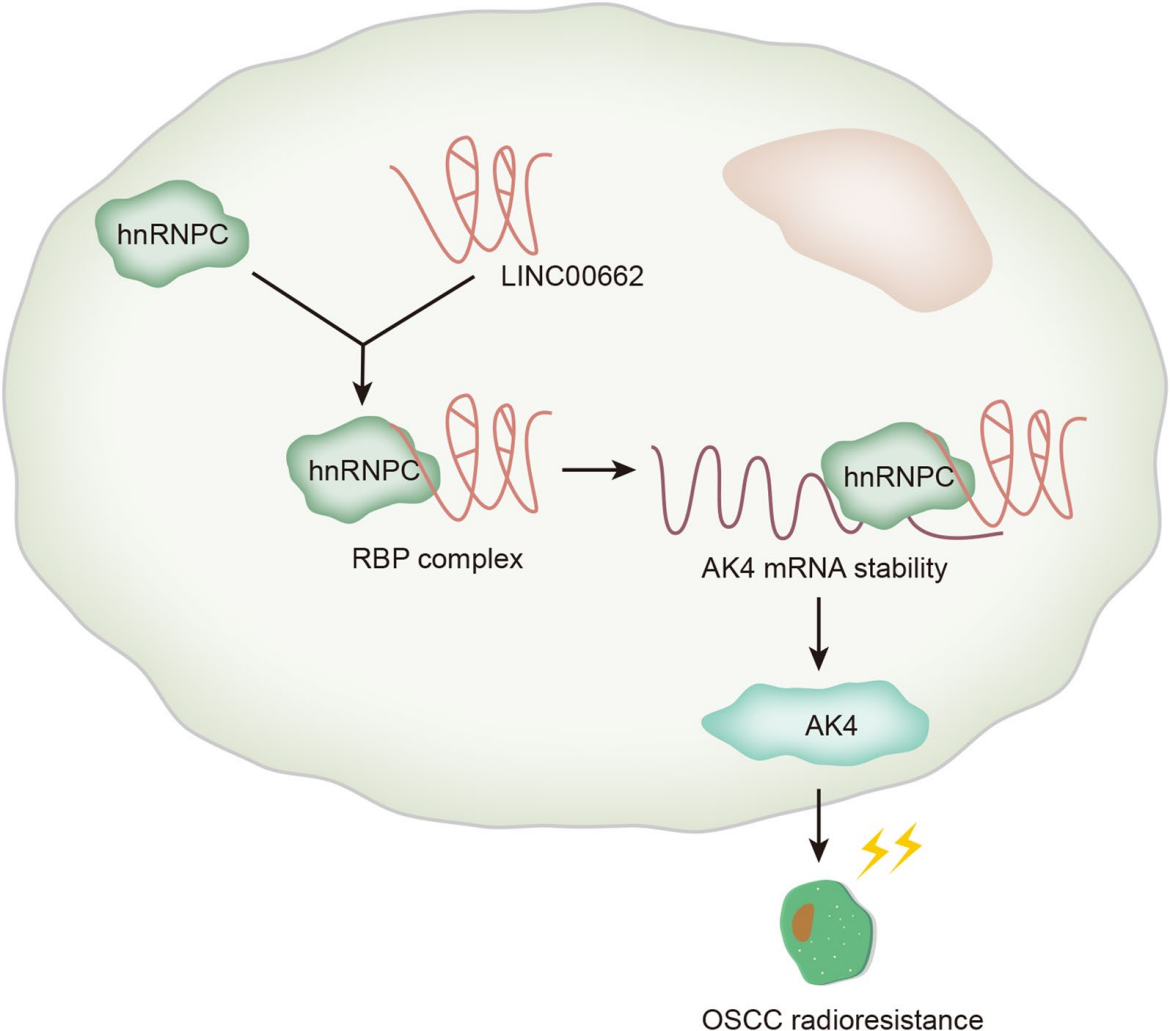
The model showing the involvement of LINC00662 in promoting radioresistance in OSCC cells: LINC00662 recruited hnRNPC protein for the binding with AK4 mRNA, which improved the mRNA stability of AK4, thus improving the radioresistance of OSCC cells

Next, the rescue assays were conducted using AK4 overexpression to rescue LINC00662 knockdown in cell radio-resistance. CCK-8 experiment demonstrated that under 4Gy irradiation, cell viability of CAL27 and SCC-4 cells was further repressed by LINC00662 knockdown but rescued by AK4 overexpression (Fig. 6a). In colony formation assay, the increasing of radiation decreased the survival fraction of CAL27 and SCC-4 cells gradually, which was reinforced by the silencing of LINC00662 but restored by the overexpression of AK4 (Fig. 6b). Under 4Gy radiation, cell cycle was arrested when LINC00662 was knocked down whereas, this effect was neutralized when AK4 was overexpressed (Additional file 4: Figure S3A). Flow cytometry analysis illustrated that under 4Gy radiation, LINC00662 down-regulation promoted the apoptosis capacity of CAL27 and SCC-4 cells, and this effect was counteracted by AK4 up-regulation (Fig. 6c). Finally, western blot disclosed that after 4Gy radiation, cleaved PARP and cleaved caspase-3 levels were also reduced after LINC00662 was knocked down but partly abolished when AK4 was overexpressed (Fig. 6d). As for cell motility, it was observed from transwell experiments that under 4Gy radiation treatment, the migration and invasion abilities were initially hampered through LINC00662 downregulation, which was repressed through AK4 promotion (Additional file 4: Figure S3B, C). Moreover, we repeated the functional rescue assays using down-regulation of AK4 to rescue LINC00662. AK4 expression was significantly down-regulated at both mRNA and protein levels by transfection of sh-AK4 (Additional file 5: Figure S4A and Additional file 1F). CCK-8 and colony formation assay depicted that silenced AK4 rescued the promoting effects of LINC00662 overexpression on cell viability and proliferation under radiation exposure (Additional file 5: Figure S4B, C). Flow cytometry cell cycle analysis revealed that, under 4Gy irradiation exposure, cell cycle arrest was inhibited by LINC00662 overexpression, and such effect was counteracted by silenced AK4 (Additional file 5: Figure S4D). Flow cytometry apoptosis assay and western analysis of apoptosis-related proteins revealed that under 4Gy irradiation exposure, silenced AK4 restored the suppressive effects of LINC00662 overexpression on cell apoptosis (Additional file 5: Figure S4E–F). Transwell assay demonstrated that silenced AK4 rescued the promoting effects of LINC00662 overexpression on cell migration and invasion under 4Gy irradiation exposure (Additional file 5: Figure S4G–H). Overall, LINC00662 served as a scaffold to recruit hnRNPC protein for stabilization of AK4, and promoted the radiosensitivity of OSCC cells via up-regulation of AK4 (Fig. 7).

## Discussion

Oral squamous cell carcinoma (OSCC) ranks as the eighth commonest oral malignancy in the world [26]. Radiotherapy is one of the main treatments for cancers, however, its therapeutic effect is limited by the radioresistance of tumors, including OSCC [27]. To solve this problem, we need to further investigate the underlying mechanism in OSCC.

Previous studies have demonstrated that lncRNAs are implicated in both physiology and pathological processes of tumors [28, 29]. The former studies have reported that LINC00662 is a newly identified oncogene and influences cellular processes in OSCC and lung cancer [13, 14]. In the present research, we found the elevated expression of LINC00662 in OSCC cell lines. In addition, the silencing or knockout of LINC00662 could promote the radiosensitivity of OSCC cells by inhibiting cell proliferation, migration and invasion, and inducing apoptosis and cell cycle arrest.

RNA-binding proteins have been unveiled to interact with lncRNAs or target genes, exerting their function in tumors. For example, A FUS (fused in sarcoma)-LATS12 axis restrains hepatocellular carcinoma via activation of Hippo pathway [30]. LncRNA SchLAH inhibits hepatocellular carcinoma metastasis via interacting with FUS [31]. LncRNA H19 boosts tumor growth and predicts a poor prognosis in colorectal cancer through recruiting eIF4A3 [32]. Here, hnRNPC is chosen for investigation. Our paper disclosed the interaction between hnRNPC protein and LINC00662. However, LINC00662 had no regulatory effects on hnRNPC expression. hnRNPC is a survival-related splicing factor in OSCC [18], and served as the tumor facilitator in non-small cell lung cancer [33], ovarian cancer [16]. The hnRNPC protein is one of the most abundant pre-mRNA-binding proteins in nucleus and has a single RNP motif RNA-binding domain [34]. hnRNPC serves as the RBP to bind the poly(U) motif and has a genome-wide effect on poly(A) site usage [35]. hnRNPC is known to stabilize uPA mRNA in the nuclear and cytosolic compartments [36]. Besides, hnRNPC was reported to interact with p53 mRNA [37] and protein [17]. Further, AK4, a radioresistant protein in esophageal cancer [20], was revealed in our study to bind with hnRNPC protein and its stability was enhanced by LINC00662. The interplay of hnRNPC protein and AK4 mRNA was firstly affirmed in our study. Overexpression of AK4 was reported to promote lung cancer metastasis [38]. AK4 was involved in hypoxia tolerance and resistance to anti-tumor drugs [39]. AK4 regulates AMPK signaling and its expression is significantly correlated with survival of glioma patients [40]. AK4 promotes lung cancer malignant progression and recurrence at an ATF3-dependent manner [21]. In our study, AK4 promoted OSCC cell proliferation, migration, invasion, and inhibited cell apoptosis and cell cycle arrest; AK4 promoted OSCC cell resistance to irritation exposure.

Moreover, lncRNAs have also been elucidated to influence cellular activities of cancers through interacting with RBPs and further affecting the expression of downstream genes [25]. For instance, lncRNA H19 demonstrates a poor prognosis of colorectal cancer and enhances tumor growth via recruiting eIF4A3 [32]. LINC00324 promotes cell proliferation of gastric cancer cells by binding to HuR and further stabilizing FAM83B expression [41]. Currently, present study uncovered that LINC00662 promoted AK4 stability by binding to hnRNPC protein, and thus exerted its pro-radioresisitance effects in OSCC cells.

## Conclusion

Finally, rescue experiments suggested that LINC00662 enhanced the radiosensitivity of OSCC cells via hnRNPC-stabilized AK4 through promoting cell proliferation, migration, invasion, and inhibiting cell apoptosis and cell cycle arrest. The current report illustrated the function role and molecular mechanism of LINC00662 in OSCC radiosensitivity, which provided a new direction in researches of tumorigenesis and progression of OSCC.

## Supplementary information

**Additional file 1** Flow cytometry analysis of transfection efficiency

**Additional file 2: Figure S1.** LINC00662 modulated the radioresistance of OSCC cells via regulation on cell cycle arrest and cell migration and invasion. (A) Survival fractions of CAL27, SCC-4 cells and NHOK cells at the indicated doses of 0, 2, 4 and 8Gy radiation were respectively determined by colony formation assay. (B–D) Cell cycle, migration and invasion capabilities were examined via flow cytometry and transwell experiments by LINC00662 silencing. *P < 0.05, **P < 0.01

**Additional file 3: Figure S2.** LINC00662 knockout reduced radioresistance of OSCC cells. (A) The expression of LINC00662 by transfection of LINC00662-KO in CAL27 and SCC-4 cells was measured by qRT-PCR. (B) CCK-8 assay was performed to examine cell viability of LINC00662-KO transfected CAL27 and SCC-4 cells under 0 or 4Gy radiation, compared with relative control groups. (C) Survival fractions of LINC00662-KO treated CAL27 and SCC-4 cells at the indicated doses of 0, 2, 4 and 8Gy radiation were respectively determined by colony formation assay. (D) Flow cytometry analysis of cell apoptosis in CAL27 and SCC-4 cells with LINC00662 knockout after 0 or 4Gy irradiation treatment. (E) Under 0 or 4Gy irradiation, cleaved PARP, cleaved caspase-3, total PARP and caspase-3 levels in CAL27 and SCC-4 cells with LINC00662 knockout were detected through western blot. (F–H) Cell cycle, migration and invasion capabilities were examined via flow cytometry and transwell experiments by LINC00662 knockout. **P < 0.01

**Additional file 4: Figure S3.** Transfection efficiency of plasmids and cell cycle, migration and invasion detection. (A–C) Cell cycle, migration and invasion capabilities were examined via flow cytometry and transwell experiments with AK4 overexpression to rescue silenced LINC00662. *P < 0.05, **P < 0.01

**Additional file 5: Figure S4.** Silenced AK4 rescued the promoting effects of LINC00662 overexpression on the radiosensitivity of OSCC cells. (A) The knockdown efficacy of AK4 in CAL27 and SCC-4 cells was detected by qRT-PCR and western blot assay. (B) CCK-8 experiment evaluated cell proliferation of CAL27 and SCC-4 cells under 4Gy irradiation with AK4 down-regulation to rescue LINC00662 overexpression. (C) In colony formation assay, survival fraction of CAL27 and SCC-4 cells was determined at the indicated doses of 0, 2, 4 and 8Gy irradiation with AK4 down-regulation to rescue LINC00662 overexpression. (D–H) Cell cycle, apoptosis, migration and invasion abilities were tested through flow cytometry, western blot and transwell assays in CAL27 and SCC-4 cells with AK4 down-regulation to rescue LINC00662 overexpression. *P < 0.05, **P < 0.01

## Data Availability

Research data and material are not shared.

## Abbreviations

OSCC: Oral squamous cell carcinoma
lncRNAs: Long non-coding RNAs
LINC00662: Long intergenic non-protein coding RNA 662
AK4: Adenylate kinase 4
hnRNPC: Heterogeneous nuclear ribonucleoprotein C
qRT-PCR: Quantitative real-time PCR
CCK-8: Cell counting kit-8
RIP: RNA immunoprecipitation
shRNA: Short hairpin RNA
FBS: Fetal bovine serum
SD: Standard deviation
ANOVA: Analysis of variance
PVDF: Polyvinylidene fluoride
SDS-PAGE: Sodium dodecyl sulfate-polyacrylamide gel electrophoresis
GAPDH: Glyceraldehyde-3-phosphate dehydrogenase
NC: Negative control
