# Supplementary figures and images for: Knockdown of LINC00662 represses AK4 and attenuates radioresistance of oral squamous cell carcinoma
Source: Cancer Cell Int. 2020 Jun 16;20:244. doi: 10.1186/s12935-020-01286-9 (PMC7296632; doi:10.1186/s12935-020-01286-9)

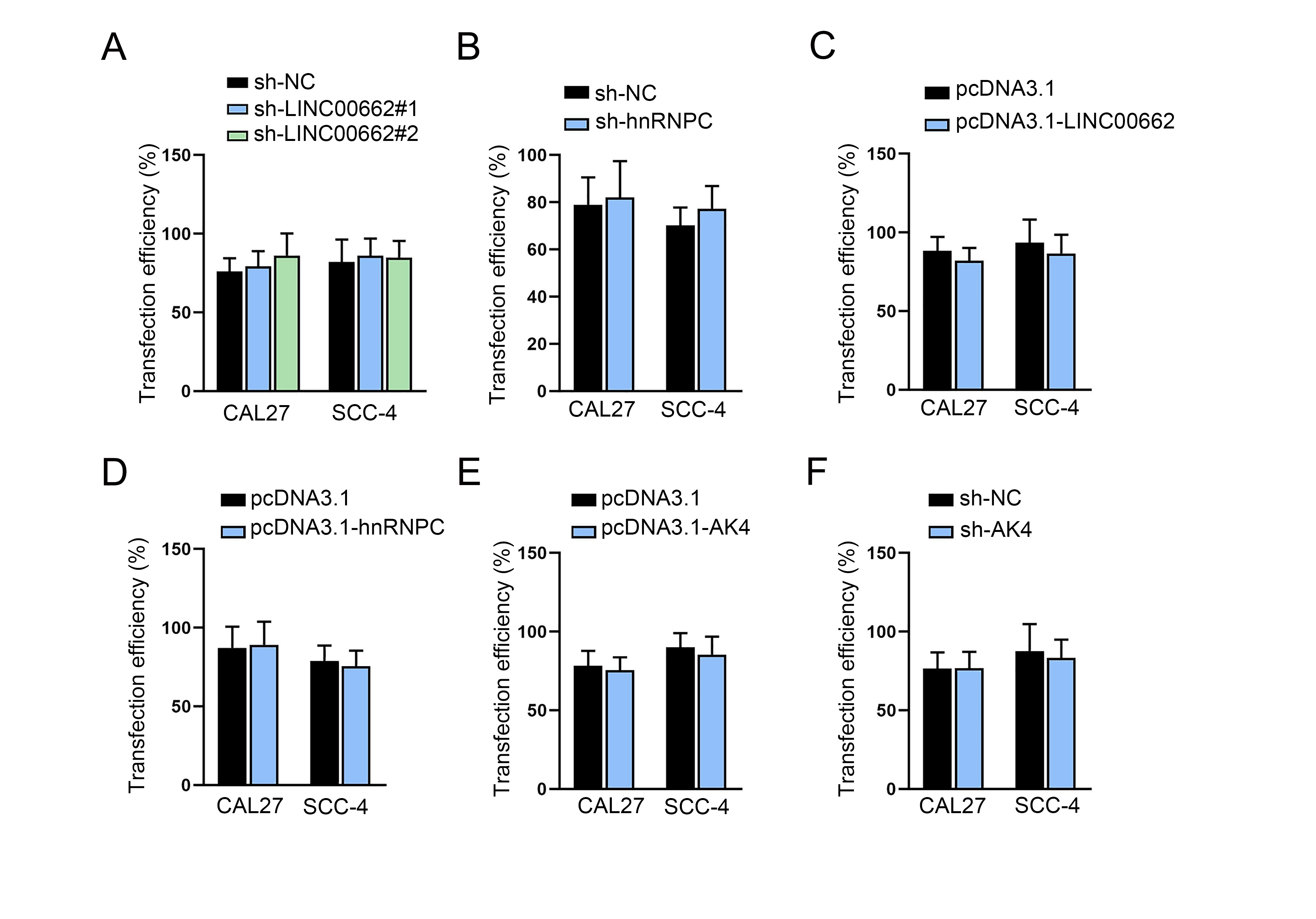

Supplement: Supplementary file 1 — Additional file 1 Flow cytometry analysis of transfection efficiency [file 12935_2020_1286_MOESM1_ESM.tif]

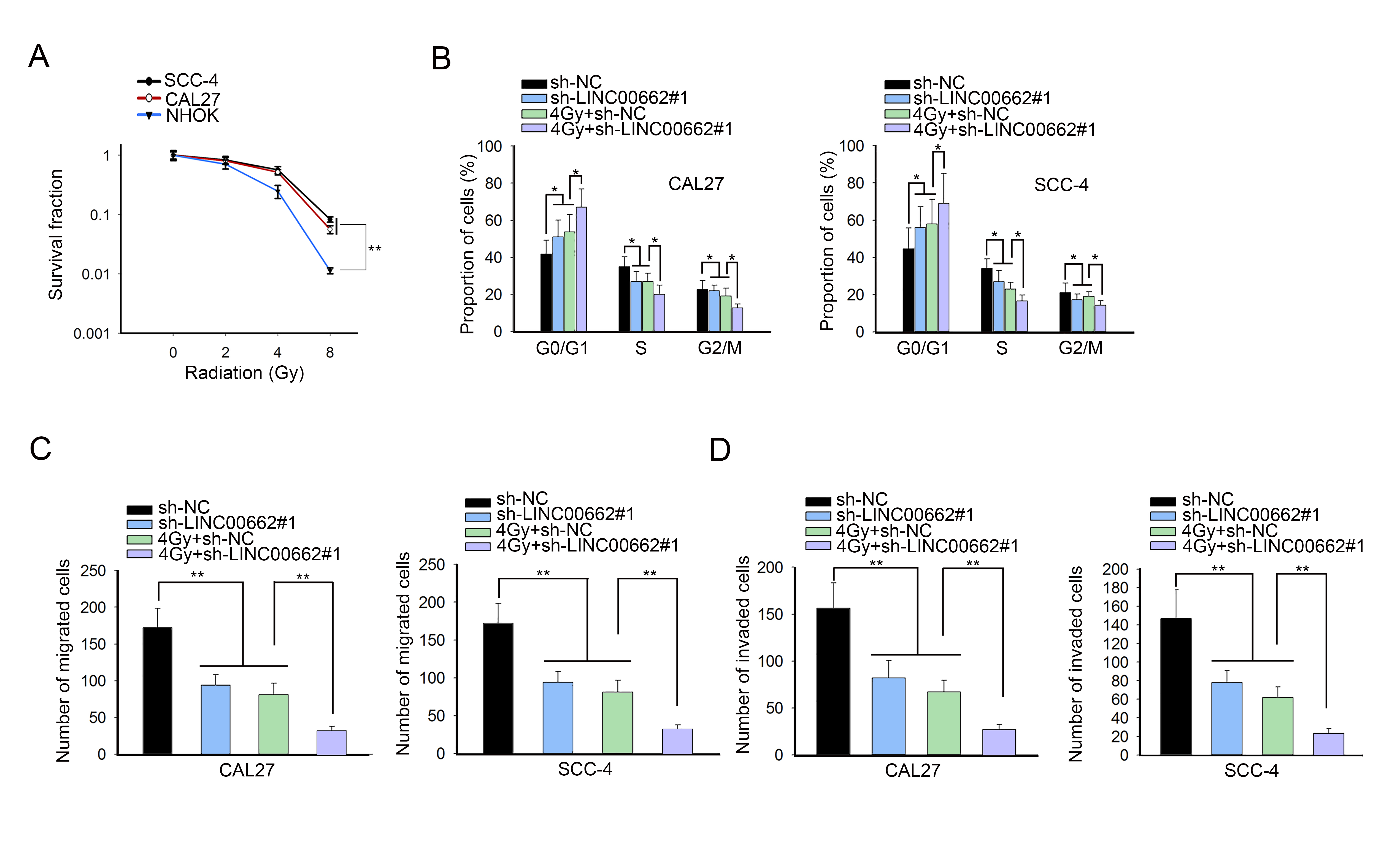

Supplement: Supplementary file 2 — Additional file 2: Figure S1. LINC00662 modulated the radioresistance of OSCC cells via regulation on cell cycle arrest and cell migration and invasion. (A) Survival fractions of CAL27, SCC-4 cells and NHOK cells at the indicated doses of 0, 2, 4 and 8Gy radiation were respectively determined by colony formation assay. (B–D) Cell cycle, migration and invasion capabilities were examined via flow cytometry and transwell experiments by LINC00662 silencing. *P < 0.05, **P < 0.01 [file 12935_2020_1286_MOESM2_ESM.tif]

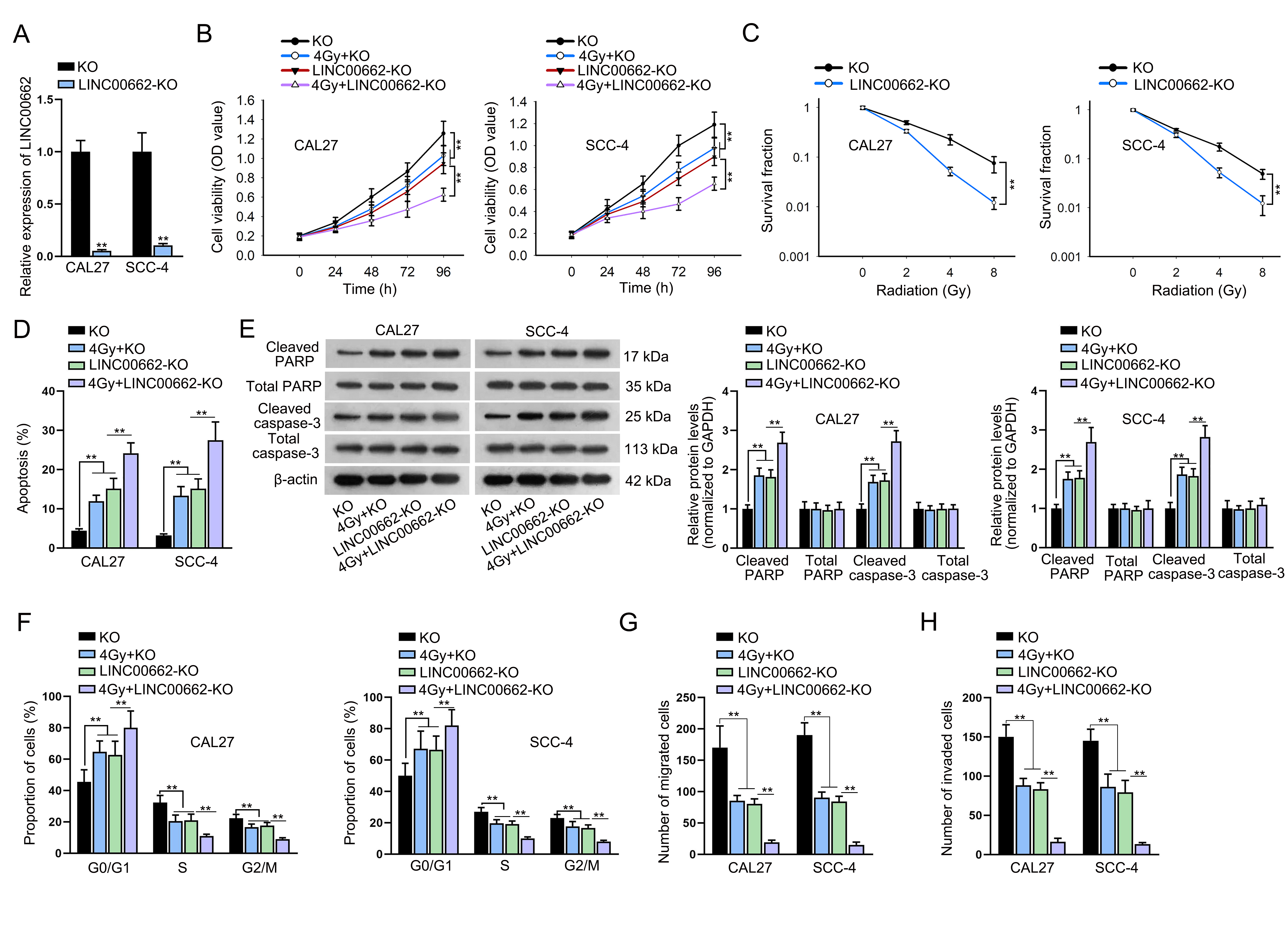

Supplement: Supplementary file 3 — Additional file 3: Figure S2. LINC00662 knockout reduced radioresistance of OSCC cells. (A) The expression of LINC00662 by transfection of LINC00662-KO in CAL27 and SCC-4 cells was measured by qRT-PCR. (B) CCK-8 assay was performed to examine cell viability of LINC00662-KO transfected CAL27 and SCC-4 cells under 0 or 4Gy radiation, compared with relative control groups. (C) Survival fractions of LINC00662-KO treated CAL27 and SCC-4 cells at the indicated doses of 0, 2, 4 and 8Gy radiation were respectively determined by colony formation assay. (D) Flow cytometry analysis of cell apoptosis in CAL27 and SCC-4 cells with LINC00662 knockout after 0 or 4Gy irradiation treatment. (E) Under 0 or 4Gy irradiation, cleaved PARP, cleaved caspase-3, total PARP and caspase-3 levels in CAL27 and SCC-4 cells with LINC00662 knockout were detected through western blot. (F–H) Cell cycle, migration and invasion capabilities were examined via flow cytometry and transwell experiments by LINC00662 knockout. **P < 0.01 [file 12935_2020_1286_MOESM3_ESM.tif]

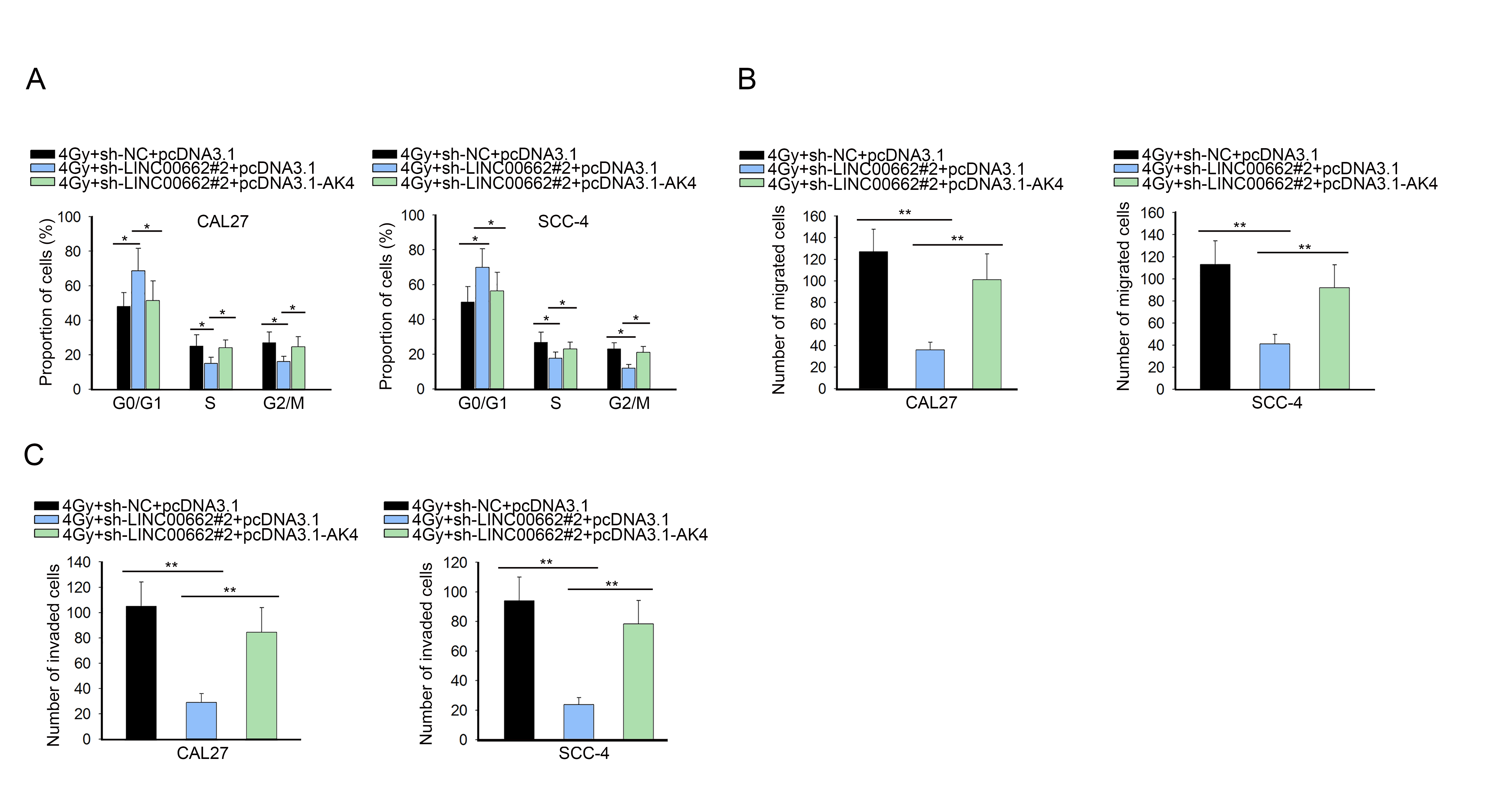

Supplement: Supplementary file 4 — Additional file 4: Figure S3. Transfection efficiency of plasmids and cell cycle, migration and invasion detection. (A–C) Cell cycle, migration and invasion capabilities were examined via flow cytometry and transwell experiments with AK4 overexpression to rescue silenced LINC00662. *P < 0.05, **P < 0.01 [file 12935_2020_1286_MOESM4_ESM.tif]

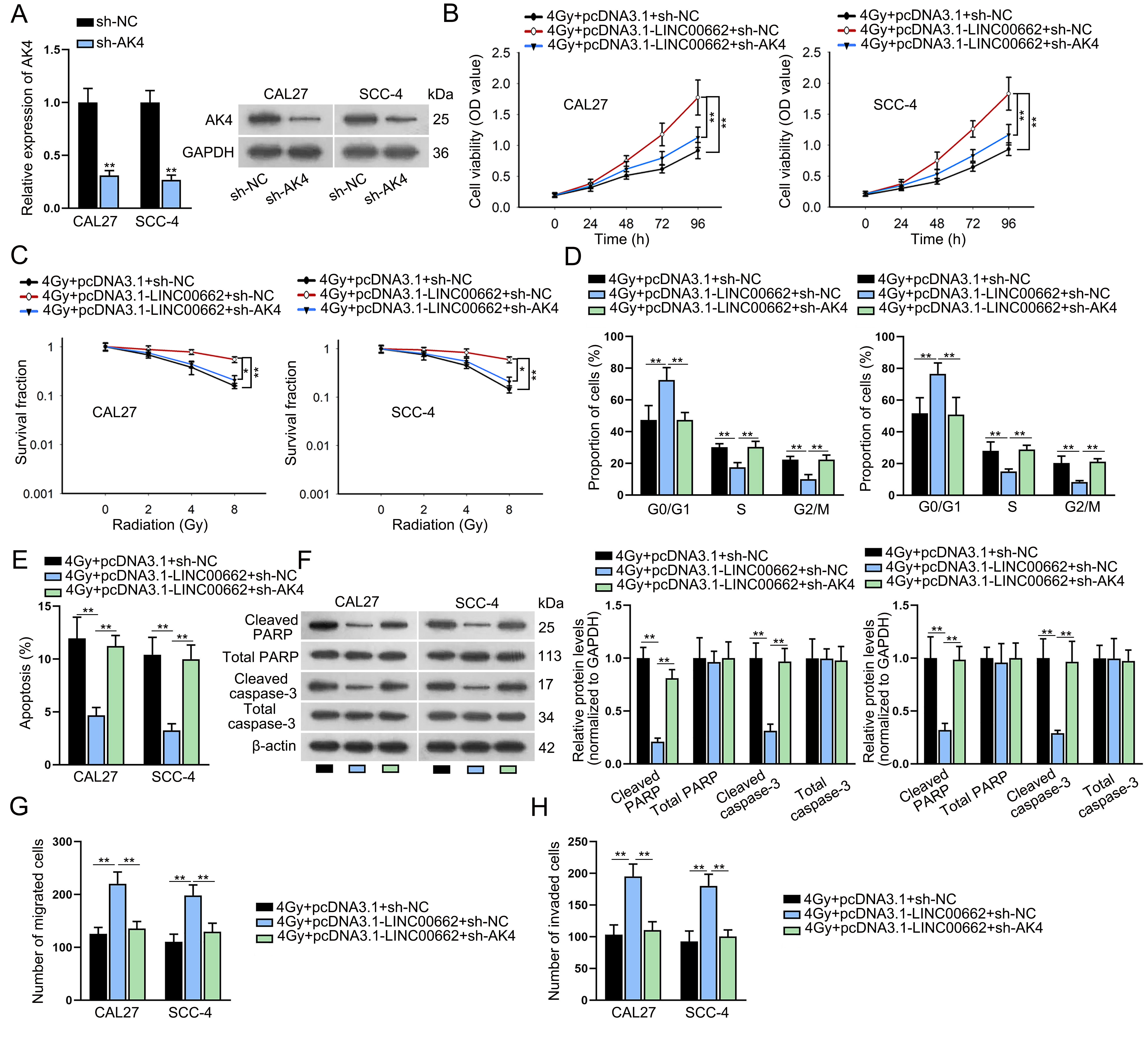

Supplement: Supplementary file 5 — Additional file 5: Figure S4. Silenced AK4 rescued the promoting effects of LINC00662 overexpression on the radiosensitivity of OSCC cells. (A) The knockdown efficacy of AK4 in CAL27 and SCC-4 cells was detected by qRT-PCR and western blot assay. (B) CCK-8 experiment evaluated cell proliferation of CAL27 and SCC-4 cells under 4Gy irradiation with AK4 down-regulation to rescue LINC00662 overexpression. (C) In colony formation assay, survival fraction of CAL27 and SCC-4 cells was determined at the indicated doses of 0, 2, 4 and 8Gy irradiation with AK4 down-regulation to rescue LINC00662 overexpression. (D–H) Cell cycle, apoptosis, migration and invasion abilities were tested through flow cytometry, western blot and transwell assays in CAL27 and SCC-4 cells with AK4 down-regulation to rescue LINC00662 overexpression. *P < 0.05, **P < 0.01 [file 12935_2020_1286_MOESM5_ESM.tif]
